# *Dehydrocostus lactone* attenuates atherogenesis by promoting cholesterol efflux and inhibiting inflammation via TLR2/PPAR-γ/NF-κB signaling pathway

**DOI:** 10.1186/s10020-025-01265-8

**Published:** 2025-06-19

**Authors:** Weitao Hong, Xiaojia Chen, Jiahai Xiao, Gengji Chen, Jiali Yang, Pengfei Zhang, Zhizhen Zhang

**Affiliations:** 1 Department of Biochemistry and Molecular Biology, School of Basic Medical Sciences, Guangdong Medical University, No. 1 Xincheng Road, Songshan Lake Sci. & Tech. Industry Park, Dongguan, Guandong 523808 China; 2 Department of Human Anatomy, School of Basic Medical Sciences, Guangdong Medical University, Dongguan, 523808 China; 3 Department of Medical Laboratory, Shenzhen Longhua District Central Hospital, Shenzhen, 518110 China

**Keywords:** Dehydrocostus lactone, Atherosclerosis, Cholesterol efflux, Anti-inflammation, Toll-like receptor 2

## Abstract

**Background:**

Dehydrocostus lactone (DHL), a natural sesquiterpene lactone, has significant anti-inflammatory effects and has the potential to inhibit ox-LDL-induced atherosclerosis in laboratory settings. However, the in vivo anti-atherosclerotic effects of DHL and their molecular mechanisms remain unclear. This study explores the anti-atherosclerosis effects of DHL on apolipoprotein E-deficient (ApoE^−/−^) mice, and the potential mechanism on macrophage-derived foam cells.

**Methods:**

Blood lipid and arterial plaque were assessed to evaluate the anti-atherosclerosis effect. The levels of inflammatory cytokines were quantified by ELISA assay. A serum metabolomics assay was performed to determine the changes in blood metabolites. A cholesterol efflux assay was used to measure the cholesterol efflux rate. Expression of genes or proteins were examined by qRT-PCR, western blot analysis, or immunofluorescence staining.

**Results:**

Treatment with DHL greatly reduced blood lipid levels and decreased the formation of atherosclerotic plaques in the aorta in high-fat diet-fed ApoE^−/−^ mice. DHL treatment enhanced cholesterol efflux from foam cells by increasing the expression of ATP-binding cassette (ABC) A1, ABCG1, and peroxisome proliferator-activated receptor gamma (PPAR-γ), both in vitro and in vivo. DHL treatment decreased the levels of IL-1β and TNF-α, elevated IL-10 levels, and promoted the formation of M2 macrophages by inhibiting myeloid differentiation factor 88 and nuclear factor kappa B (NF-κB). Inhibition of TLR2 in foam cells derived from macrophages significantly reduced the inflammatory response and enhanced cholesterol efflux.

**Conclusion:**

This study demonstrates that treatment with DHL alleviates atherosclerosis by promoting cholesterol efflux and inhibiting inflammation through the TLR2/PPAR-γ/NF-κB signaling pathway.

**Graphical abstract:**

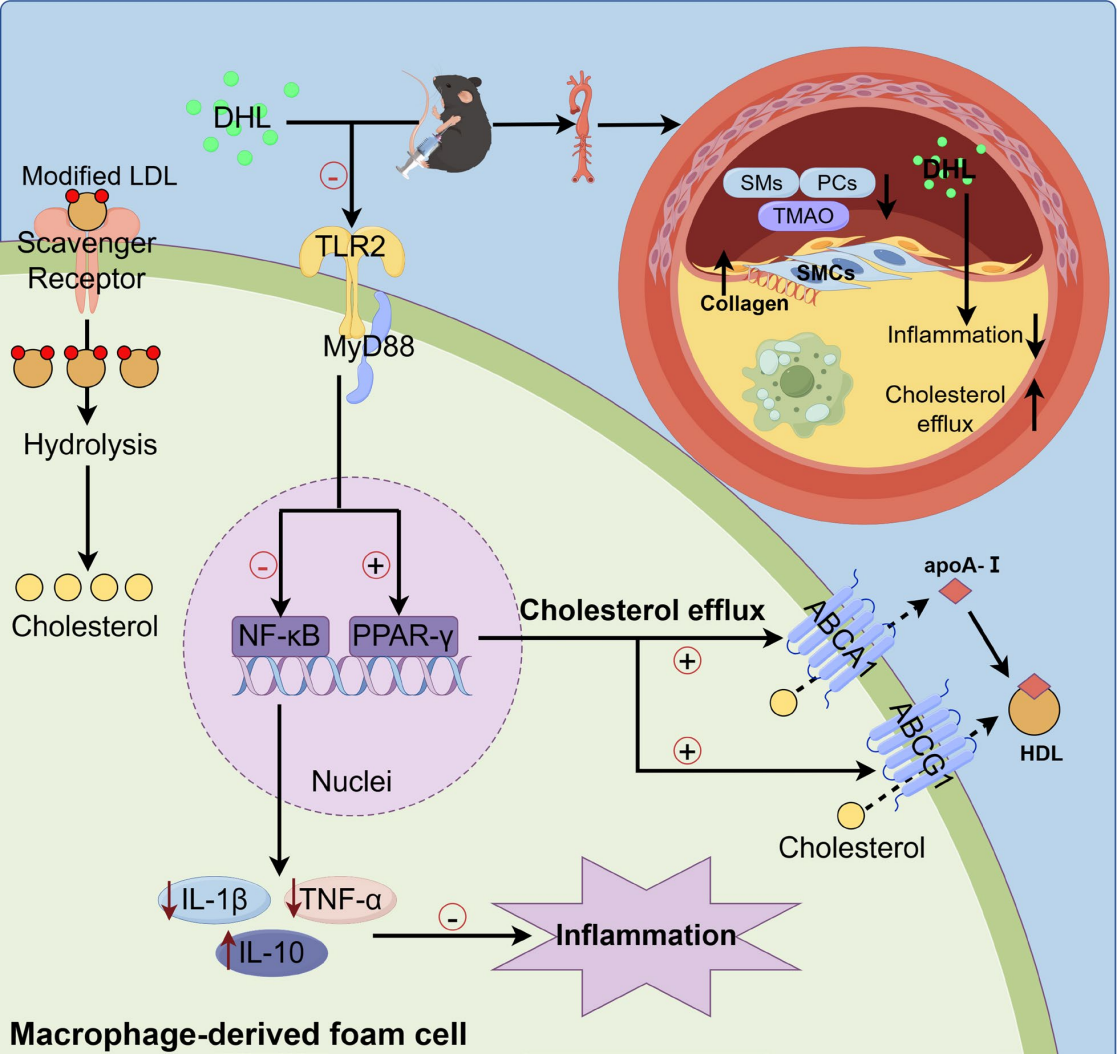

## Introduction

Atherosclerosis (AS) is the underlying pathophysiological process of cardiovascular disease. It is a chronic inflammatory disease resulting from abnormalities in lipid metabolism (Kobiyama and Ley, 2018; Libby 2021a). In AS, the impaired function of endothelial cells causes the deposition of low-density lipoprotein (LDL) in the arterial wall. The LDL then undergoes modifications, such as oxidization to form oxidized LDL (ox-LDL) or acetylation to form acetylated LDL (ac-LDL). The excessive accumulation of lipids recruits a significant number of monocytes into the intima, where they differentiate into macrophages and efficiently internalize and engulf LDL particles, producing foam cells (Allahverdian et al. 2018; Fernández-Ruiz 2022). Foam cells release inflammatory factors, reactive oxygen species, and other mediators that amplify local inflammation, resulting in cell death and the formation of necrotic lipid cores within AS plaques (Kong et al. 2022; Soehnlein and Libby 2021). The formation of foam cells is a characteristic feature of atherosclerotic lesions, regulated by a delicate balance between lipid uptake, cholesterol esterification, and cholesterol efflux (Wang et al. 2019a, b). Consequently, targeting these three primary pathways could be an effective therapeutic strategy for treating atherosclerosis.

Foam cells effectively remove cholesterol through ATP-binding cassette A1 (ABCA1) and ATP-binding cassette G1 (ABCG1) (Westerterp et al. 2014). ABCA1 facilitates free cholesterol excretion to apolipoprotein A-I (apoA-I) and nascent high-density lipoprotein (HDL) particles, while ABCG1 enhances free cholesterol loading onto more mature HDL particles (Frambach et al. 2020). The deletion of ABCA1 and ABCG1 in macrophages accelerates the development of atherosclerosis in LDL receptor-knockout mice, underscoring the pivotal role of cholesterol efflux mediated by ABCA1 and ABCG1 in preventing atherosclerosis (Ouimet et al. 2019). Moreover, inflammation mediated by macrophage-derived foam cells significantly contributes to the formation, progression, and rupture of atherosclerotic plaques. Consequently, targeting inflammatory pathways holds promise for the prevention and treatment of atherosclerosis (Kong et al. 2022; Soehnlein and Libby 2021). Toll-like receptors (TLRs), widely expressed in innate immune cells, play important roles in innate and adaptive immune responses. TLRs, including TLR2 and TLR4, have been implicated in inflammation and the development of atherosclerotic plaques (Jin et al. 2023). Activation of TLRs suppresses the activity of peroxisome proliferators-activated receptors (PPARs) and liver X receptor (LXR), hampering cholesterol efflux from foam cells and potentially exacerbating TLR recruitment and inflammation (Hsieh et al. 2020; Tall and Yvan-Charvet 2015). Thus, inhibition of TLRs may have dual effects, reducing inflammation and promoting cholesterol efflux, making it a potential therapeutic strategy.

Dehydrocostus lactone (DHL) is a natural sesquiterpene lactone derived from *Aucklandia lappa Decne*. Its biological properties include anti-inflammatory, antibacterial, antioxidant, anti-allergic, and anti-tumor effects (Deyno et al. 2020; Li et al. 2020a, b; Pyun et al. 2018; Xiong et al. 2021). Recent studies have indicated that DHL has the ability to prevent ox-LDL-induced atherosclerosis by inhibiting the adhesion of monocytes to endothelial cells (Wang et al. 2019a, b). Furthermore, DHL has been shown to significantly reduce M1 polarization and enhance M2 polarization in macrophages challenged with gram-positive bacteria, achieved through the inhibition of toll-like receptor signaling pathways (Kim et al. 2020; Wu et al. 2021). Consequently, DHL may have the potential to inhibit the formation of foam cells derived from macrophages and prevent atherosclerosis. The focus of this study was to investigate the effect of DHL on lipid accumulation and inflammation in apolipoprotein E-deficient (ApoE^−/−^) mice. The potential mechanism of action for DHL was explored by assessing the rate of cholesterol efflux, anti-inflammatory effects, and the activation of the toll-like receptor pathway. This research aims to establish an empirical basis for utilizing DHL as a treatment for atherosclerosis.

## Materials and methods

### Drugs preparation


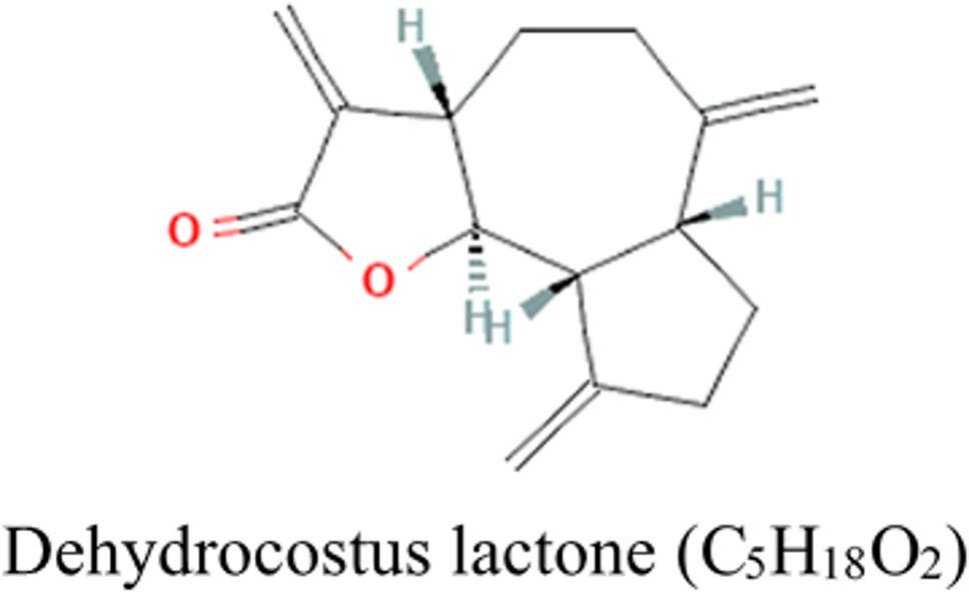


Dehydrocostus lactone (DHL) with a purity of 98% (catalog number: B21026; HPLC analysis) was obtained from Shanghai Yuanye Bio-Technology Co., Ltd (Shanghai, China). The molecular formula of DHL is shown in the figure on the right. Simvastatin (catalog number: S831014; 98% purity, HPLC analysis) was purchased from Shanghai Macklin Biochemical Technology Co., Ltd (Shanghai, China).

Before the treatment, DHL and Simvastatin were dissolved in a mixed vehicle solution composed of 10% dimethyl sulfoxide (DMSO), 40% PEG300, 5% Tween-80, and 45% saline to obtain the stock concentrations of 20 mg/mL and 60 mg/mL, respectively.

### Animals and treatment

Wild-type (WT) mice and ApoE^−/−^ mice (6–8 weeks old, weighing 20–25 g) were obtained from GemPharmatech Co., Ltd. (Strain ID: T001458, Nanjing, China). All animals were housed under controlled conditions, maintaining a temperature of 24 ± 2℃, a humidity level of 50 ± 5%, and a standard 12-hour light/dark cycle. The animal facility ensured a pathogen-free environment. A total of 48 mice (8 WT mice and 40 ApoE^−/−^ mice) were randomized into the control group (WT mice fed a standard chow diet), the AS group (ApoE^−/−^ mice fed a high-fat diet consisting of 89% basal diet, 10% lard oil, and 1% cholesterol) with vehicle solution treatment, AS with low concentration of DHL (10 mg/kg/day, L-DHL group) treatment, AS with a moderate concentration of DHL (20 mg/kg/day, M-DHL group) treatment, AS with a high concentration of DHL (40 mg/kg/day, H-DHL group) treatment, and the simvastatin group (6 mg/kg/day). Each group comprised 8 mice. The mice were fed a high-fat diet for 8 weeks to induce atherosclerosis, followed by a 10-week treatment with DHL and simvastatin via intraperitoneal injection at every 2 days (3:00 p.m.). The injection dosage was calculated according to the drug concentration and individual animal weight. Mice were provided with ad libitum access to food and water throughout the experiment.

### Blood lipid determination

Approximately 1 mL of whole blood samples was obtained from the angular vein of anesthetized mice using pentobarbital. Serum samples were obtained by centrifugating the whole blood at 1790 × *g* for 10 min at room temperature. Then, the serum samples were stored in microtubes at −80 °C. The serum levels of triglyceride (TG), total cholesterol (TC), high-density lipoprotein cholesterol (HDL-C), and low-density lipoprotein cholesterol (LDL-C) were measured using commercially available kits purchased from Applygen Technologies Inc. (E1003, E1005, E1017, and E1018; Beijing, China).

### Aortic plaques assessment

Mice were euthanized by cervical dislocation in accordance with ethical guidelines. The entire aorta was carefully dissected, and the adipose tissue surrounding the arterial structure was thoroughly removed. Then, they were stored in a 4% paraformaldehyde solution in PBS at room temperature for 24 h. The oil red O staining was performed to assess the plaque level of the whole aorta. In brief, the aorta was longitudinally cut along the vessel wall and soaked in oil red O solution (G1016, Servicebio, Wuhan, China) for 60 min. Subsequently, it was differentiated using 75% ethanol until the fatty plaques in the lumen appeared orange or bright red, while the other parts became nearly colorless. Finally, the aortas were rinsed twice with distilled water.

### Analysis of atherosclerotic plaques in aortic root

The aortas were fixed in 4% paraformaldehyde in PBS for 24 h at room temperature. Following fixation, the aortas were dehydrated in a 30% sucrose solution and subsequently embedded in an optimal cutting temperature compound. Each sample was then sectioned into 5 slices, with each slice containing 3 sections of 8 μm thickness from aortic root. Hematoxylin and eosin (H&E) staining was performed on the sections, which were later sealed with neutral resin for analysis of arterial morphological changes. Lipid content in the plaques was visualized analysis using oil red O staining, while Masson staining was employed to assess the collagen content. The level of smooth muscle cells was determined by immunohistochemical staining of alpha-smooth muscle actin (α-SMA) using a rabbit polyclonal antibody (pAb, GB111364, Servicebio, Wuhan, China). For analysis of macrophage polarization in the plaques, immunofluorescence staining was carried out using anti-CD68 rabbit pAb (GB113109, Servicebio, Wuhan, China) and anti-CD163 pAb (GB113751, Servicebio, Wuhan, China). Finally, the CaseViewer software was utilized to calculate the positive area ratio of the slides (Yang et al. 2025).

### Determination of cytokine levels in serum and foam cells

The serum concentrations of interleukin (IL)−1β, IL-10, and tumor necrosis factor-α (TNF-α) were determined using ELISA kits (EMC001bQT, EMC005, and EMC102a; NeoBioscience Technology Co., Ltd., Shenzhen, China).

The levels of IL-1β, IL-10, and TNF-α in the culture supernatant were quantified using ELISA kits. Detection of absorbance at 450 nm was performed using a microplate reader. Concentrations of TNF-α, IL-1β, and IL-10 in each group were calculated using the standard curve.

### Serum metabolomics assay

Eighteen serum samples collected from WT group (6 samples), AS group (6 samples), and H-DHL group (6 samples) were submitted for metabolomics analysis. Serum metabolomic profiling was conducted in collaboration with Gene Denovo Biotechnology Co., Ltd. (Guangzhou, China), which encompassed mass spectrometry analyses and/or bioinformatics analysis. Multivariate statistical analysis was performed following the processing and annotation of the original LC-MS data. Discrimination of metabolites between the two sample classes was achieved by applying a statistically significant threshold, notably the Variable Importance in Projection (VIP) value (VIP > 1), which was further validated through Student’s t-test analysis (*P* ≤ 0.05). For the comparison of groups, the Orthogonal Projection to Latent Structures-Discriminant Analysis (OPLS-DA) was employed utilizing R package models. Additionally, to explore alternative metabolic pathways, differential metabolites underwent grouping and enrichment analysis using MetaboAnalyst 5.0 in conjunction with the KEGG database.

### Quantitative RT-PCR analysis

Total RNA samples were isolated from the aorta and macrophages using Trizol reagent (15596026, Invitrogen™, Carlsbad, CA, USA). Subsequently, a reverse transcription was performed on 500 ng of total RNA in a 10 µL system to synthesize cDNA, employing the PrimeScript™ RT reagent Kit with gDNA Eraser (RR047 A, Takara, Beijing, China). Quantitative PCR (qPCR) was conducted to analyze the expressions of target genes, utilizing SYBR Green I dye under the following conditions: 95 °C for 30 s, 95 °C for 5 s, 60 °C for 34 s, with 40 cycles, followed by 95 °C for 15 s, 60 °C for 1 min, and 95 °C for 15 s for one cycle. The primer sequences used for the target genes, including TLR2, TLR4, myeloid differentiation factor 88 (MyD88), nuclear factor kappa B (NF-κB), PPAR-γ, LXR, ABCA1, ABCG1, IL-1β, IL-10, TNF-α, and glyceraldehyde-3-phosphate dehydrogenase (GAPDH) are listed in Table 1. Relative mRNA expressions of the target genes compared to GAPDH were calculated utilizing the 2^−∆∆CT^ method.

**Table 1:** Primer sequences used for real-time RT-qPCR

Gene name	Primer sequence (5′−3′)
TLR2 (NM_011905.3）	Forward-TCCCAGATGCTTCGTTGTTCCC
	Reverse-AGTGGTTGTCGCCTGCTTCC
TLR4 (NM_021297.3)	Forward-GAATGAGGACTGGGTGAGAAATGAG
	Reverse-TGGATGATGTTGGCAGCAATGG
MyD88 (NM_010851.3)	Forward-GCAGAACCAGGAGTCCGAGAAG
	Reverse-GATGCCTCCCAGTTCCTTTGTTTG
NF-κB (NM_001365067.1)	Forward-TGGAAGGCTCATGGTTGGATGTG
	Reverse-AGTGACTTTATGGGAACCCGATGG
PPAR-γ (NM_001127330.3)	Forward-TGTTCGCCAAGGTGCTCCAG
	Reverse-TGAAGGCTCATGTCTGTCTCTGTC
LXR (NM_001177730.1)	Forward-TTCTTCCGCCGCAGTGTCATC
	Reverse-TTTCCGCCGCATGTAGGTGTC
ABCA1 (NM_013454.3)	Forward-AGGCTGCTGCTGTGGAAGAATC
	Reverse-ATGTTGTTCGTAGGGTGGGTAGC
ABCG1 (NM_009593.2)	Forward-GCTGCTGCCTCACCTCACTG
	Reverse-CTCTCGTCTGCCTTCATCCTTCTC
IL-1β (NM_008361.4)	Forward-CTCGCAGCAGCACATCAACAAG
	Reverse-CCACGGGAAAGACACAGGTAGC
IL-10 (NM_010548.2)	Forward-GGTTGCCAAGCCTTATCGGAAATG
	Reverse-GCCGCATCCTGAGGGTCTTC
TNF-α (NM_001278601.1)	Forward-CCACCACGCTCTTCTGTCTACTG
	Reverse-TGGTTTGTGAGTGTGAGGGTCTG
GAPDH (NM_001289726.2)	Forward-AGGTCGGTGTGAACGGATTTG
	Reverse-GGGGTCGTTGATGGCAACA

### Immunohistochemistry

Immunohistochemistry was employed to assess the expression of TLR2 (GB11518, Servicebio, Wuhan, China) and MyD88 (GB111554, Servicebio Wuhan, China) in the aortic root. The slides were prepared by paraffin-embedding, slicing, and subsequent deparaffinization. Antigen retrieval was performed by heating the slides in a microwave oven for 20 min in a 0.1 mol/L sodium citrate solution, followed by treatment with 1% hydrogen peroxide to eliminate endogenous peroxidase activity. The slides were then blocked with 2% goat serum for 1 h. Primary antibodies were applied at a dilution of 1:200 in goat serum and incubated overnight at 4℃. This was followed by incubation with horseradish peroxidase conjugated goat anti-rabbit immunoglobulin G (1:500; A0208, Beyotime Biotechnology, Shanghai, China) for 2 h at room temperature. Nuclei were counterstained with hematoxylin for 5 min. The slides were mounted with coverslips and visualized under a light microscope (magnification, ×500; Nikon Corporation). ImageJ 1.45 software (US National Institutes of Health, Bethesda, MD, USA) was utilized for analysis.

### Macrophage’s culture and foam cell formation

Mouse RAW264.7 cells (CL-0190, Pricella Biotechnology Co., Ltd, Wuhan, China) and human monocytic THP-1 cells (CL-0233, Pricella Biotechnology Co., Ltd, Wuhan, China), were cultured in a 37 °C incubator with 5% CO_2_. RAW264.7 cells were maintained in DMEM high-glucose media, while THP-1 cells were maintained in RPMI 1640 high-glucose media, both supplemented with 10% fetal bovine serum and 1% penicillin/streptomycin (100 units/mL penicillin and 100 µg/mL streptomycin). To induce the differentiation of THP-1 monocytes into macrophages, 160 nmol/L PMA was utilized. The formation of macrophage-derived foam cells was achieved by incubating the cells with a serum-free medium (DMEM or RPMI 1640) containing 0.2% bovine serum albumin (BSA) and 50 µg/mL acetylated-low density lipoprotein (ac-LDL; JK-011; Anhui Jingke Biotechnology Co., LTD, Anhui, China) for 48 h.

### Cholesterol efflux assay

To determine cholesterol efflux, RAW264.7 macrophages (1 × 10^4^ cells/well) and THP-1 macrophages (1.5 × 10^4^ cells/well) were seeded into 24-well plates. The cells were then incubated with a serum-free medium consisting of 0.2% BSA, 2 µg/mL NBD-cholesterol (810250P, Avanti Research™, Alabama, United States), and 50 µg/mL ac-LDL for 48 h. Following this, the cells were incubated with a serum-free DMEM (or RPMI 1640) consisting of 0.2% BSA for 12 h to balance the intracellular lipid. Finally, the cells were incubated with varying concentrations (0, 1, 5, and 10 µmol/L) of DHL in serum and phenol red-free DMEM (or RPMI 1640) medium for 12 h to induce cholesterol efflux. The levels of NBD-cholesterol in cells and medium were measured using a microplate reader by detecting excitation light at a wavelength of 465 nm and emission light at 535 nm. Cholesterol efflux (%) was calculated by dividing the fluorescence intensity in the efflux media by the total fluorescence intensity (media plus cells) and multiplying the result by 100%.

To quantify cellular cholesterol levels, foam cells derived from macrophages were subjected to a 12-hour treatment with or without 5 µmol/L DHL. TC level was measured using an assay kit (E1015, Applygen Technologies, Beijing, China). Protein concentration in the same lysate was determined using the Enhanced BCA Protein Assay Kit (P0009, Beyotime Biotechnology, Shanghai, China). Total cellular cholesterol was reported as nmol/mg protein, with results presented as the mean ± standard deviation of three independent experiments. Oil red O staining was employed to assess lipid droplet levels in the cells, and images were captured using an inverted light microscope (Axio Vert. A1, Carl Zeiss, Jena, Germany).

### Western blot analysis

To validate the lipid-lowering and anti-inflammatory properties of DHL, the cells were incubated with 0, 1, 5, and 10 µmol/L of DHL in the presence or absence of TLR2 agonist Pam3 CSK4 (tlrl-pm2 s-1, Invivogen™, Carlsbad, CA, USA) and the TLR2 inhibitor C29 (HY-100461, MedChem Express, Shanghai, China).

Total protein samples were collected by extracting cells in RIPA lysis buffer with a protease inhibitor (P1006, Beyotime Biotechnology, Shanghai, China). The resulting mixture was then centrifuged at 12,000 × *g* for 10 min at 4 ℃. Subsequently, 30 µg of total proteins per lane were separated using 8%, 10%, or 12% SDS-PAGE, depending on the size of the target proteins. These proteins were then electrophoretically transferred to polyvinylidene difluoride (PVDF) membranes in a trans-buffer at 100 V for 1 or 2 h. For blocking, PVDF membranes were treated with either 5% skimmed milk or 5% bovine serum albumin (for phosphorylated proteins) in TBST buffer (20 mM Tris-HCl, pH 7.5, 150 mM sodium chloride, and 0.05% Tween 20) for 2 h. After three washes with TBST buffer for 5 min each, the membranes were incubated overnight with primary antibodies against MyD88 (4283 S, Cell Signaling Technology, Shanghai, China), NF-κB (8242 S, Cell Signaling Technology, Shanghai, China), PPAR-γ (ab272718, Abcam, Shanghai, China), LXR (sc-377260, Santa cruz, Texas, USA), ABCA1 (ab66217, Abcam, Shanghai, China), ABCG1 (MA5-35185, Thermo, Shanghai, China), and GAPDH (2118 S, Cell Signaling Technology, Shanghai, China). Subsequently, the membranes were incubated with anti-rabbit (A0208, Beyotie Biotechnology, Shanghai, China) or anti-mouse (A0216, Beyotime Biotechnology, Shanghai, China) secondary antibodies conjugated to horseradish peroxidase (1:2000) at room temperature for 2 h. Protein bands were detected using enhanced chemiluminescence and quantified with Fiji software (W. Rasband, NIH, USA), with GAPDH as the normalizer.

### Molecular docking

To analyze the binding affinities and modes of interaction between DHL and TLR2, AutodockVina 1.2.2, a silico protein-ligand docking software was employed (Morris et al. 2008; Xu et al. 2024). The molecular structure of DHL (PubChem CID: 73174) was retrieved from PubChem Compound (Wang et al. 2017a, b) (https://pubchem.ncbi.nlm.nih.gov/). The 3D coordinate of TLR2 (PDB ID: 6 NIG; resolution, 2.35 Å) was downloaded from the PDB (http://www.rcsb.org/pdb/home/home.do). For docking analysis, all protein and molecular files were converted into PDBQT format with all water molecules excluded and polar hydrogen atoms were added. The grid box was centered to cover the domain of each protein and allow for unrestricted molecular movement. The grid box was set to 30 Å × 30 Å × 30 Å, and grid point distance was 0.05 nm. Molecular docking studies were performed by Autodock Vina 1.2.2 (http://autodock.scripps.edu/).

### Statistical analysis

Quantitative data are reported as mean ± SD from the independent experiments. Statistical analysis was performed using Prism 9 (GraphPad, San Diego, CA, USA) software. The multiple comparisons were conducted by one-way ANOVA with Tukey’s test. Significance was defined as *P* < 0.05.

## Results

### DHL treatment decreases blood lipid levels and atherosclerotic plaques and promotes plaque stability in atherosclerotic mice

**Fig. 1:**
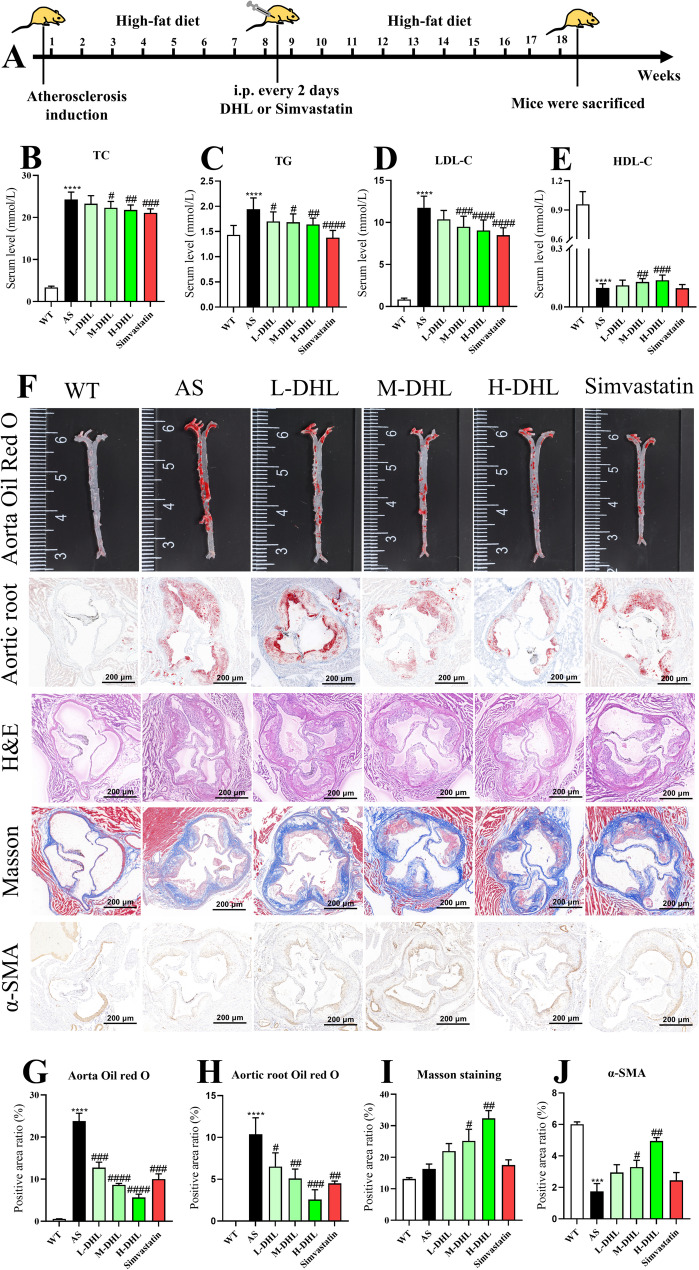
Effect of DHL on blood lipid level and atherosclerotic plaque development in ApoE^−/−^ mice. **A** Schematic overview of the in vivo experiment. **B** Serum total cholesterol (TC) level. **C** Serum triglycerides (TG) level. **D** Serum low-density lipoprotein cholesterol (LDL-C) level. **E** Serum high-density lipoprotein cholesterol (HDL-C). **F** Representative images of aortic arches, oil red O staining of en-face analysis of aortic lesions in the entire aorta (First row); cross-sections of the aortic root stained with oil red O staining (Second row); H&E staining of aortic root (Third row); Masson staining of aortic root (Fourth row); immunohistochemical staining of α-SMA in aorta root (Fifth row). **G** Quantification of atherosclerotic plaques is shown as a percentage of the whole aorta. **H** Quantification of lipid content in aorta root. **I** Quantification of collagen content in aorta root. **J** Quantification of vascular smooth muscle. Data are expressed as mean ± SD (*n* = 8). *P < 0.05, **P < 0.01, ***P < 0.001 (*AS group vs. WT group). ^#^*P* < 0.05, ^##^*P* < 0.01, ^###^*P* < 0.001 (^#^DHL and Simvastatin treatments vs. AS group). WT: wide type; AS: atherosclerosis; DHL: dehydrocostus lactone

ApoE^−/−^ mice were fed a high-fat diet (HFD) for eight weeks to expedite atherosclerosis formation, followed by a ten-week treatment with DHL or Simvastatin (Fig. 1A). Compared to the AS group, mice treated with DHL exhibited a significant decrease in serum levels of TC (Fig. 1B), triglycerides (TG) (Fig. 1C), and low-density lipoprotein cholesterol (LDL-C) (Fig. 1D) (all *P* < 0.05). Furthermore, DHL treatment led to an increase in serum levels of high-density lipoprotein cholesterol (HDL-C) (Fig. 1E) (*P* < 0.05). Oil red O staining of lipid droplets in the entire artery (Fig. 1F, first row) and aortic root (Fig. 1F, second row) indicated a reduction in lipid accumulation with DHL treatment (Fig. 1G and H) (*P* < 0.05) in both regions. H&E staining of the aortic root (Fig. 1F, third row) supported the presence of smaller plaques in the DHL-treated mice. Masson staining of the aortic root (Fig. 1F, fourth row) demonstrated a significant increase in collagen content in the DHL groups compared to the AS group (Fig. 1I) (*P* < 0.05), reflecting enhanced arterial plaque stability. Immunohistochemical staining of α-SMA in the aortic root (Fig. 1F, fifth row) further revealed a significant increase in vascular smooth muscle cells with DHL treatment (Fig. 1J) (*P* < 0.05). Moreover, simvastatin therapy significantly reduced serum lipid levels (TC, TG, and LDL-C) and arterial plaque content but did not affect the serum level of HDL-C.

### DHL treatment inhibits inflammation in atherosclerotic mice

**Fig. 2:**
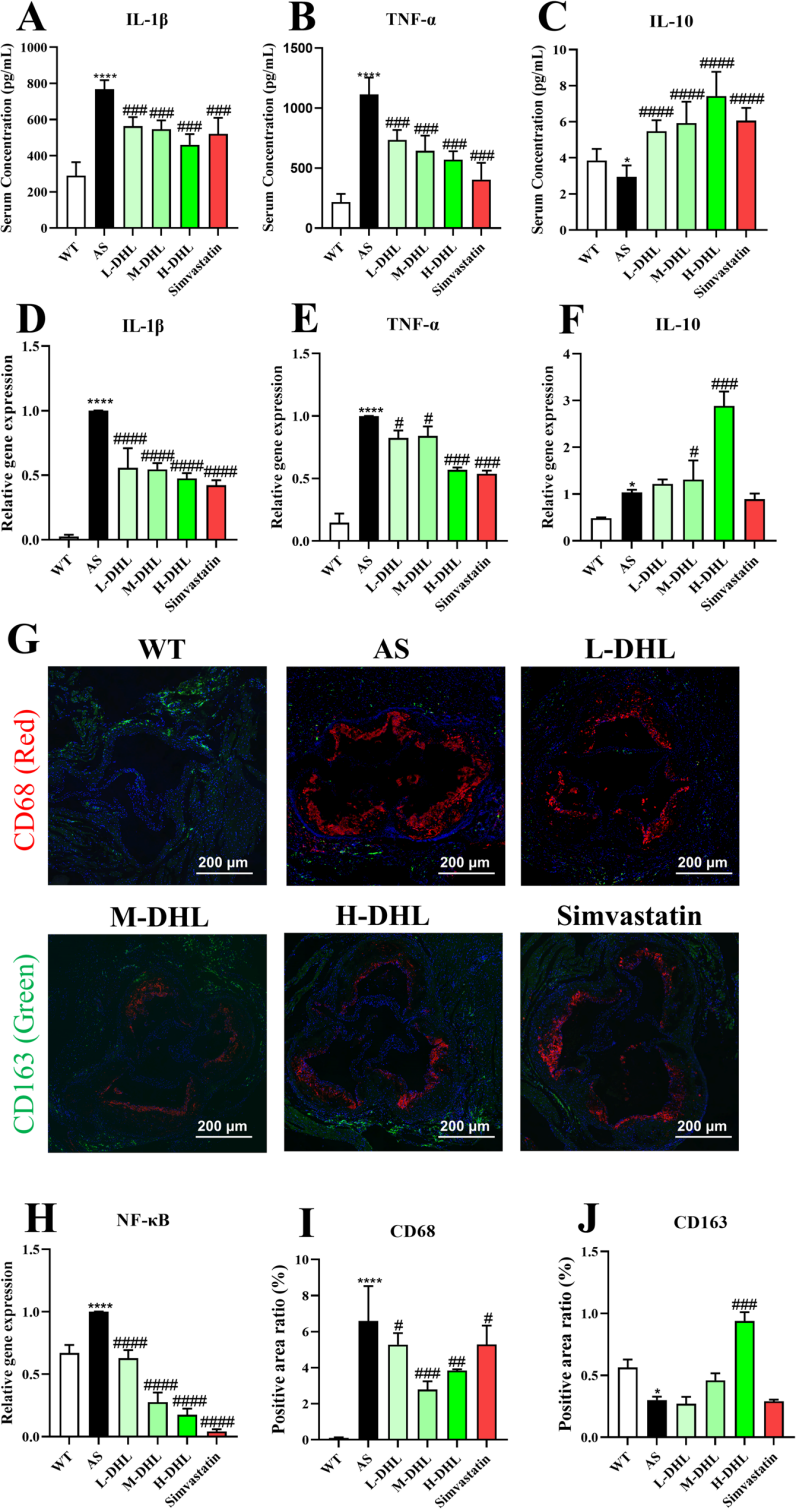
Anti-inflammatory effect of DHL. **A-C** Serum cytokines level of IL-1β (A), TNF-α (**B**), IL-10 (**C**). **D-E** The gene expression of IL-1β (**D**), TNF-α (**E**), IL-10 (**F**). **G** Representative images of immunofluorescence staining of CD68 (Marker of M1 macrophage) and CD163 (Marker of M2 macrophage) in the aorta root. **H** The gene expression of IL-1β. **I** Quantification of M1 macrophages in aorta root. **J** Quantification of M2 macrophages in aorta root. Data are expressed as mean ± SD (*n* = 8). *P < 0.05, **P < 0.01, ***P < 0.001 (*AS group vs. WT group). ^#^*P* < 0.05, ^##^*P* < 0.01, ^###^*P* < 0.001 (^#^DHL and Simvastatin treatments vs. AS group). WT: wide type; AS: atherosclerosis; DHL: dehydrocostus lactone

The elevation of proinflammatory cytokines contributes to the progression of atherosclerosis. The results of the current study revealed that mice in the AS group exhibited higher serum levels of IL-1β and TNF-α, while demonstrating lower serum levels of IL-10 compared to the WT group. Treatment with DHL substantially reduced the levels of IL-1β (Fig. 2A) and TNF-α (Fig. 2B) in the serum, while concurrently increasing the levels of IL-10 (Fig. 2C) (all *P* < 0.05). Similarly, DHL treatment resulted in decreased gene expression of IL-1β (Fig. 2D) and TNF-α (Fig. 2E) in the aorta, accompanied by an increase in the expression of IL-10 in the aorta (Fig. 2F) (all *P* < 0.05). The release of cytokines was regulated by NF-κB, the results showed that mice in the AS group exhibited higher expression of NF-κB in the aorta compared to the WT group (*P* < 0.05), but treatment with DHL significantly decreased the gene expression of NF-κB (Fig. 2H) (*P* < 0.05). Macrophage infiltration, a crucial indicator of vascular inflammation and foam cell formation, was evaluated through immunofluorescence staining of CD68 for M1 macrophages and CD163 for M2 macrophages (Fig. 2G). The findings demonstrated that the content of M1-macrophages (CD68 staining) at the root of the aorta was significantly higher in the AS group than in the WT group (*P* < 0.05). Conversely, the content of M2-macrophages (CD163 staining) was notably lower (*P* < 0.05). Treatment with DHL markedly reduced the positive area ratio of CD68 (Fig. 2I). and increased the positive ratio of CD163 (Fig. 2J) (all *P* < 0.05). Furthermore, simvastatin therapy substantially reduced the levels of IL-1β, TNF-α, and macrophage infiltration, while concurrently increasing the level of IL-10 (all *P* < 0.05).

### DHL treatment inhibits blood choline metabolism and sphingolipid metabolism

**Fig. 3:**
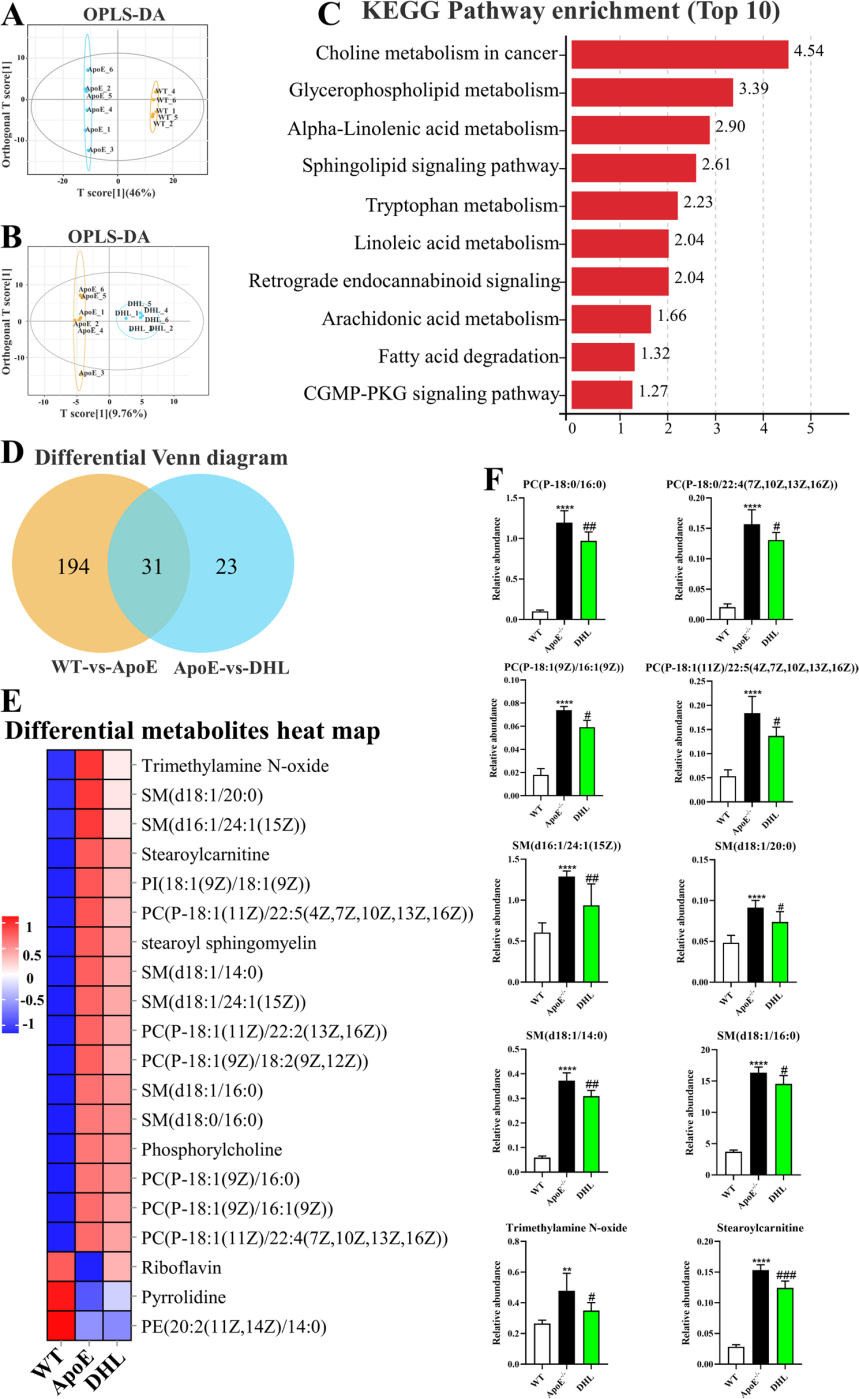
DHL treatment inhibits blood choline metabolism and sphingolipid metabolism. **A** OPLS-DA score plots for the samples remarkably separated the ApoE group from the WT groups. **B** OPLS-DA score plots for the samples remarkably separated the DHL group from the ApoE groups. **C** Pathway enrichment analysis showed the pathways most significantly altered among the three groups. **D** Differential Venn diagram showed the common metabolites. **E** Heat map of the differentially expressed metabolites related to choline and sphingolipid metabolism. **F** The biomarkers of 10 metabolites were selected from the common metabolites. The metabolite profiling was performed in mouse blood. Data are expressed as mean ± SD (*n* = 6). *P < 0.05, **P < 0.01, ***P < 0.001 (*ApoE group vs. WT group). ^#^*P* < 0.05, ^##^*P* < 0.01, ^###^*P* < 0.001 (^#^DHL vs. ApoE group). WT: wide type; ApoE: atherosclerosis group; DHL: dehydrocostus lactone

Eighteen blood samples were analyzed, consisting of six samples, each from the WT, AS, and H-DHL groups. The OPLS-DA score plots (Fig. 3A and B) indicated distinctive differences among the WT, ApoE^−/−^, and H-DHL groups, suggesting the potential selection of metabolic biomarkers. An analysis of metabolic pathway enrichment revealed significant enrichment in “choline metabolism in cancer,” “glycerophospholipid metabolism,” “alpha-linolenic acid metabolism,” “sphingolipid signaling pathway,” and “tryptophan metabolism” (Fig. 3C). The differential Venn diagram identified thirty-one metabolites in the three groups (Fig. 3D). Among these, twenty metabolites associated with choline metabolism or sphingolipid metabolism exhibited differential expression across the three groups (Fig. 3E). In our search for potential diagnostic biomarkers related to atherosclerosis, we observed a significant increase in the levels of four phosphatidylcholines (PC): P-18:0/16:0, P-18:0/22:4 (7Z, 10Z, 13Z, 16Z), P-18:1 (9Z)/16:1 (9Z), and P-18:1 (11Z)/22:5 (4Z, 7Z, 10Z, 13Z, 16Z); four sphingomyelins (SM): d16:1/24:1 (15Z), d18:1/20:0, d18:1/14:0, and d18:1/16:0; trimethylamine-N-oxide; and stearoyl carnitine in the ApoE^−/−^ group compared to the WT group. However, treatment with DHL significantly decreased the relative abundance of these metabolites (Fig. 3F, *P* < 0.05).

### DHL treatment promotes cholesterol efflux from macrophage-derived foam cells

**Fig. 4:**
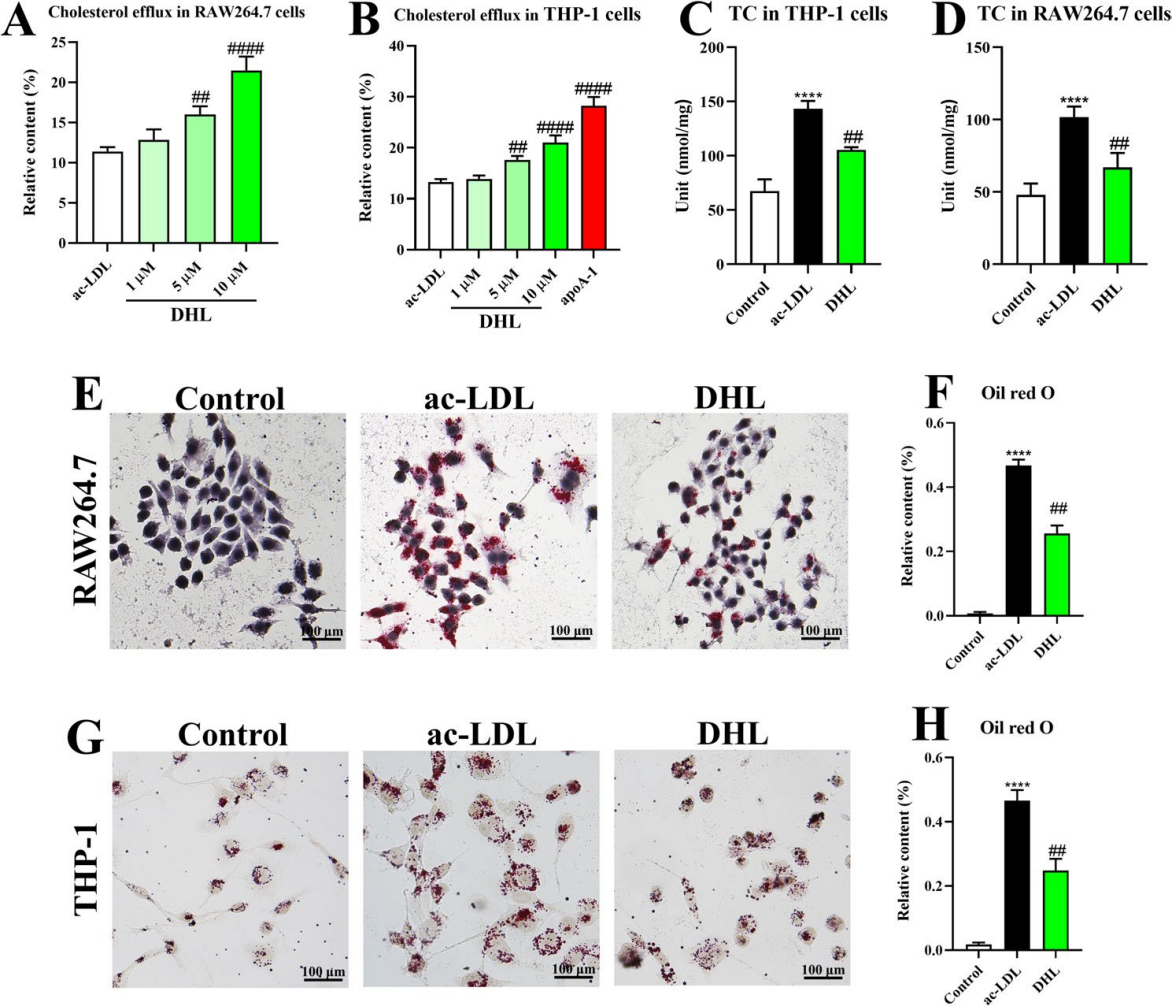
DHL treatment promotes cholesterol efflux from macrophage-derived foam cells. **A** Cholesterol efflux determination in RAW264.7 macrophages. **B** Cholesterol efflux determination in THP-1 cells. **C** Total cholesterol (TC) level in THP-1 cells. **D** Total cholesterol (TC) level in RAW264.7 macrophages. **E** Representative images of oil red O staining in RAW264.7 macrophages. **F** Quantification of lipid droplets in RAW264.7 macrophages. **G** Representative images of oil red O staining in THP-1 cells. (H) Quantification of lipid droplets in THP-1 cells. Data are expressed as mean ± SD (*n* = 6). *P < 0.05, **P < 0.01, ***P < 0.001 (*ac-LDL group vs. control group). ^#^*P* < 0.05, ^##^*P* < 0.01, ^###^*P* < 0.001 (^#^DHL vs. ac-LDL group). ac-LDL: acetylated-low density lipoprotein; DHL: dehydrocostus lactone

The prevention and treatment of AS are primarily focused on cholesterol efflux within foam cells. Thus, we investigated the effects of DHL on cholesterol efflux in RAW264.7 macrophages and THP-1 macrophage-derived foam cells. Our cholesterol efflux assay revealed that incubation with DHL significantly enhanced cellular cholesterol efflux in a concentration-dependent manner in both RAW264.7 macrophages (Fig. 4A) and THP-1 macrophage-derived foam cells (Fig. 4B) (all *P* < 0.05). To further assess the lipid-lowering activity of DHL, we measured the intracellular TC content in macrophage-derived foam cells. As depicted in Fig. 4C, D and a significant reduction in TC content was observed when cells were incubated with 5 µM of DHL for 12 h (all *P* < 0.05). Moreover, we performed oil-red O staining to visualize intracellular lipid accumulation in foam cells. The results showed that treatment with 5 µM of DHL significantly attenuated lipid accumulation in foam cells compared to the ac-LDL group (Fig. 4E, F and G, and 4H).

### DHL enhances the expression of PPAR-γ, LXR, ABCA1, and ABCG1 to promote cholesterol efflux

**Fig. 5:**
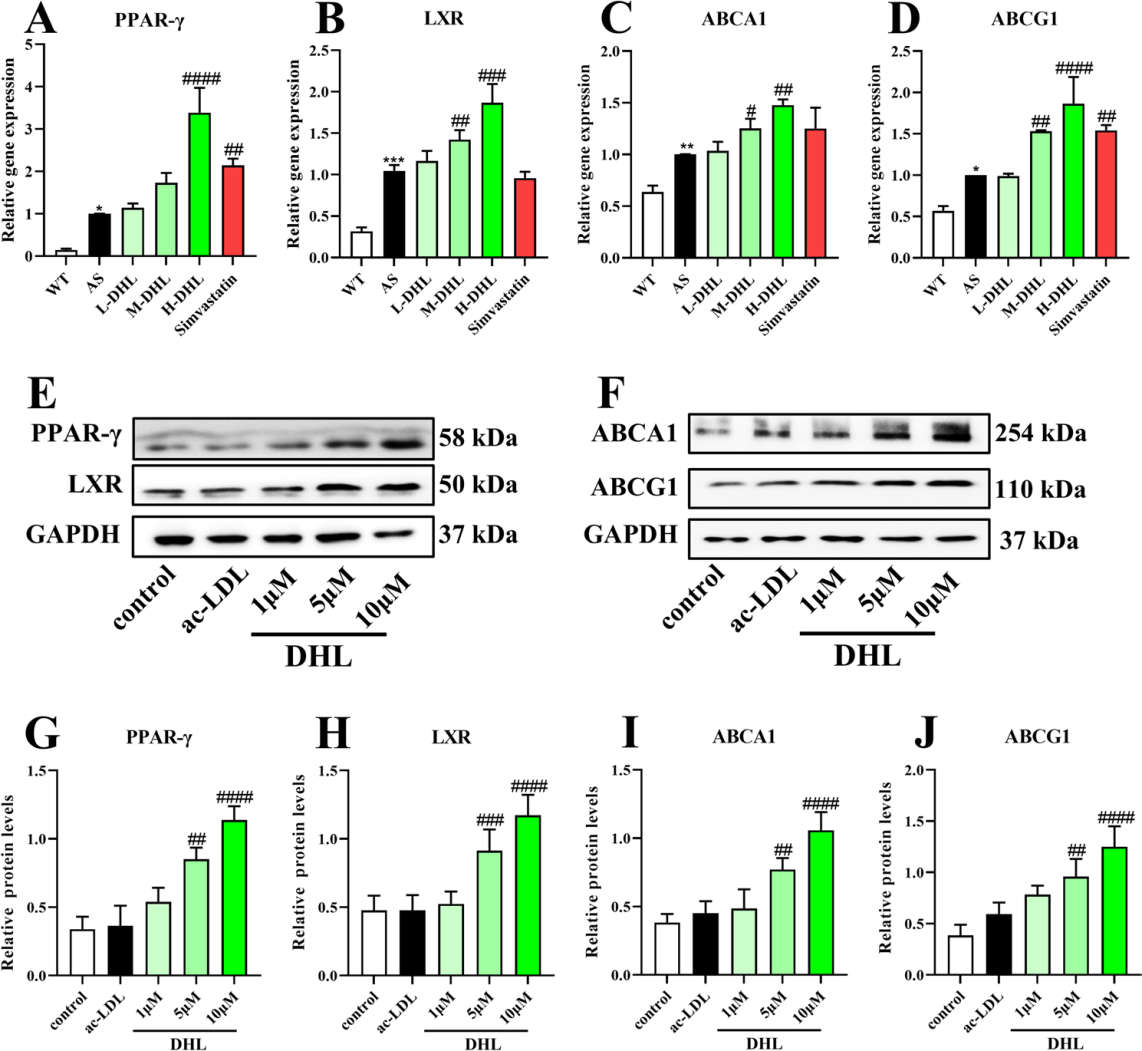
DHL enhances the expression of PPAR-γ, LXR, ABCA1, and ABCG1 in macrophage-derived foam cells. **A-D** The gene expression of PPAR-γ (**A**), LXR (**B**), ABCA1 (**C**), and ABCG1 (**D**). **E** Immunoblotting image of PPAR-γ, LXR, and GAPDH. **F** Immunoblotting image of ABCA1, ABCG1, and GAPDH. **G** The relative protein expression of PPAR-γ. **H** The relative protein expression of LXR. **I** The relative protein expression of ABCA1. **J** The relative protein expression of ABCG1. Data are expressed as mean ± SD (*n* = 6). ^#^*P* < 0.05, ^##^*P* < 0.01, ^###^*P* < 0.001 (^#^DHL and Simvastatin treatments vs. ac-LDL group). ac-LDL: acetylated-low density lipoprotein; DHL: dehydrocostus lactone

ABCA1 and ABCG1 are key proteins responsible for transporting excessive lipids out of cells, while PPAR-γ and LXR are transcription factors that promote the expression of ABCA1 and ABCG1. DHL treatment notably increased the gene expression of PPAR-γ (Fig. 5A), LXR (Fig. 5B), ABCA1 (Fig. 5C), and ABCG1 (Fig. 5D) in the aorta of atherosclerotic mice (all *P* < 0.05). A western blot was performed to assess the protein expression of PPAR-γ, LXR, ABCA1, and ABCG1 (Fig. 5E and F) in RAW264.7 cells. As depicted in Fig. 5G, H and I, and 5J, treatment with DHL resulted in a dose-dependent increase in the protein expression of PPAR-γ, LXR, ABCA1, and ABCG1 (all *P* < 0.05).

### DHL treatment inhibits atherosclerotic plaque growth via toll-like receptors

**Fig. 6:**
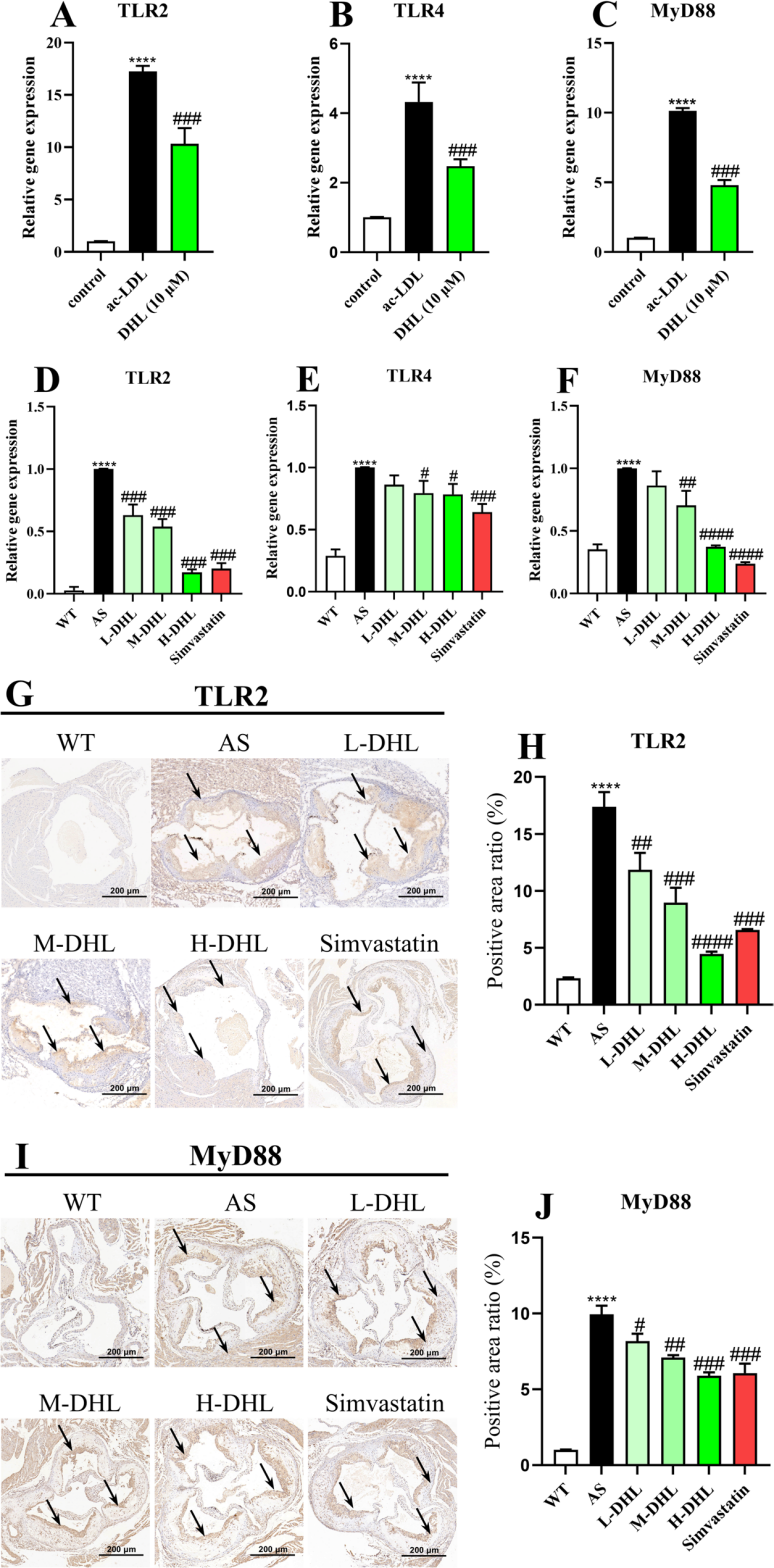
DHL treatment inhibits atherosclerotic plaque growth via toll-like receptors. **A-C** Relative gene expressions of TLR2 (**A**), TLR4 (**B**), and MyD88 (**C**) in macrophage-derived foam cells. **D-F** Relative gene expressions of TLR2 (**A**), TLR4 (**B**), and MyD88 (**C**) in the aortas of mice. **G** Immunohistochemical staining of TLR2 and (**H**) Quantification of TLR2 expression in aorta root. **I** Immunohistochemical staining of MyD88 and (**J**) Quantification of MyD88 expression in aorta root. Data are expressed as mean ± SD (*n* = 6 for cells, *n* = 8 for tissues). *P < 0.05, **P < 0.01, ***P < 0.001 (*AS group vs. WT group or ac-LDL group vs. Control group). ^#^*P* < 0.05, ^##^*P* < 0.01, ^###^*P* < 0.001 (^#^DHL and Simvastatin treatments vs. AS group or DHL group vs. ac-LDL group). WT: wide type; AS: atherosclerosis; DHL: dehydrocostus lactone

To investigate the underlying pathways responsible for the observed changes in DHL-treated atherosclerosis, we analyzed the expression of proteins associated with inflammation and cholesterol efflux in macrophage-derived foam cells and the aortas. As shown in Fig. 6, DHL potently decreased ac-LDL-induced augmentation of TLR2 (Fig. 6A), TLR4 (Fig. 6B), and MyD88 (Fig. 6C) expression at mRNA levels (all *P* < 0.05). The expression of TLR2, TLR4, and MyD88 was significantly elevated in the aorta of mice in the AS group compared to those in the WT group. However, treatment with DHL resulted in a significant decrease in the gene expression of TLR2 (Fig. 6D), TLR4 (Fig. 6E), and MyD88 (Fig. 6F) (all *P* < 0.05). Furthermore, DHL’s inhibitory effect on TLR2 is more pronounced in both arterial tissues and cells. Immunohistochemical staining of TLR2 (Fig. 6G and H) and MyD88 (Fig. 6I and J) in the aortic root also revealed that DHL treatment significantly decreased the expression of these two proteins (all *P* < 0.05).

### TLR2 is involved in the positive effects of DHL on cholesterol efflux and the anti-inflammation effect in foam cells

**Fig. 7:**
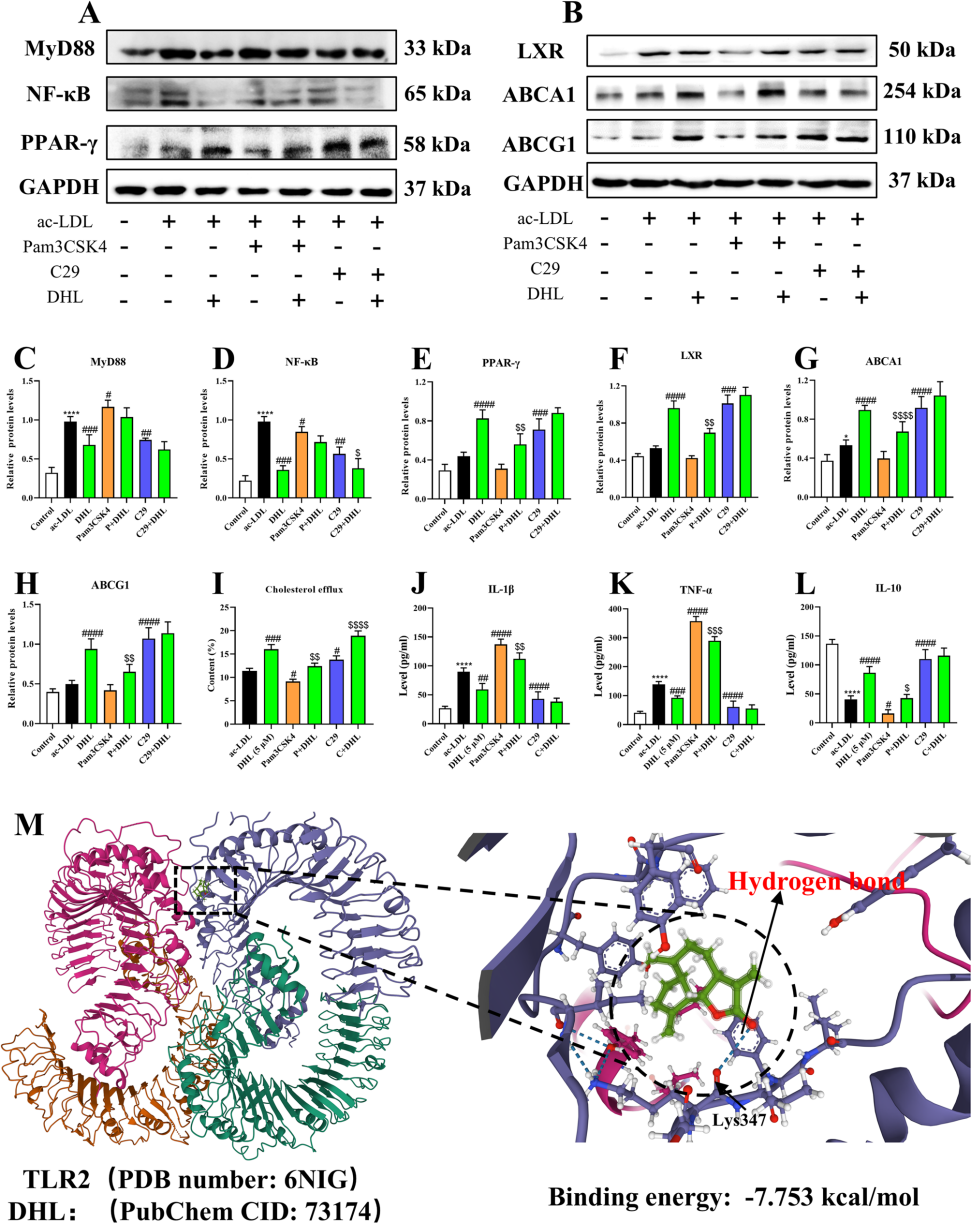
TLR2 is involved in the positive effects of DHL on cholesterol efflux and the anti-inflammation effect in foam cells. RAW264.7 macrophages were pre-treated with 50 µg/mL ac-LDL for 48 h. Then cells were treated with or without 5 µM of DHL for 12 h in the presence or absence of Pam3 CSK4 and C29 to examine whether TLR2 is involved in the effects of DHL on anti-inflammation and cholesterol efflux. **A** Immunoblotting image of MyD88, NF-κB, PPAR-γ, and GAPDH. **B** Immunoblotting image of LXR, ABCA1, ABCG1, and GAPDH. **C-H** Relative protein expressions of MyD88 (**C**), NF-κB (**D**), PPAR-γ (**E**), LXR (**F**), ABCG1 (**G**), and ABCA1 (**H**). **I** The cholesterol efflux rate in macrophage-derived foam cells. **J-L** ELISA assay was used to evaluate IL-1β (**J**), TNF-α (**K**), and IL-10 (**L**) levels in the medium. (M) Binding mode of DHL to TLR2 by molecular docking. Data are expressed as mean ± SD (*n* = 6). *P < 0.05, **P < 0.01, ***P < 0.001 (*ac-LDL group vs. control group). ^#^*P* < 0.05, ^##^*P* < 0.01, ^###^*P* < 0.001 (^#^DHL and Simvastatin treatments vs. ac-LDL group). ^$^*P* < 0.05, ^$$^*P* < 0.01, ^$$$^*P* < 0.001 (^$^DHL treatments vs. agonist or inhibitor group). ac-LDL: acetylated-low density lipoprotein; DHL: dehydrocostus lactone

To substantiate the role of TLR2 inhibition in cholesterol efflux and its anti-inflammatory effects, we employed TLR2 inhibitors (C29) and agonists (Pam3 CSK4). The immunoblotting assay was used to determine the protein expression (Fig. 7A and B). In the presence of the TLR2 inhibitor (C29), DHL treatment had no impact on the protein expression of MyD88 (Fig. 7C) (*P* > 0.05) but significantly reduced the protein expression of NF-κB (Fig. 7D) (*P* < 0.05). Conversely, In the presence of TLR2 agonists (Pam3 CSK4), DHL treatment significantly increased the protein expression of PPAR-γ (Fig. 7E), LXR (Fig. 7F), ABCA1 (Fig. 7G), and ABCG1 (Fig. 7H) (all *P* < 0.05). Moreover, the cholesterol efflux assay demonstrated that the rate of cholesterol efflux decreased after Pam3 CSK4 treatment compared to the ac-LDL group, whereas it increased after C29 treatment (Fig. 7I). In the case of DHL treatment, the efflux rate in the Pam3 CSK4 + DHL group was significantly lower than that in the DHL group (*P* < 0.05), while it was higher in the C29 + DHL group compared to the DHL group (Fig. 7I) (*P* < 0.05). Regarding cytokine levels, DHL treatment decreased the levels of IL-1β (Fig. 7J) and TNF-α (Fig. 7K), while concurrently increasing the level of IL-10 (Fig. 7L) (all *P* < 0.05) in the presence of Pam3 CSK4, but had no impact on these cytokine levels in the presence of C29. Furthermore, we conducted molecular docking analysis to evaluate the affinity between DHL and TLR2 (Figs. 7M). The results showed that DHL binds to DHL through strong electrostatic interactions and visible hydrogen bonds with the oxygens of Lys 347. Moreover, For TLR2, DHL had a low binding energy of −7.753 kcal/mol (Fig. 7M).

## Discussion

Foam cell formation and its ectopic deposition in the vascular wall are considered the distinctive features of atherosclerotic lesions. This process is regulated by the balance between lipid uptake, esterification, and efflux, as well as influenced by inflammation (Wang et al. 2019a, b). Natural products present a promising approach for inhibiting foam cell formation, thereby exerting potent anti-atherosclerosis activity (Wang et al. 2020; Zhi et al. 2023). A previous study has demonstrated that DHL has an anti-inflammatory effect and might ameliorate ox-LDL-induced atherosclerosis (Wang et al. 2019a, b). However, the in vivo anti-atherosclerotic effect of DHL and its underlying mechanism remains unclear.

In this study, ApoE^−/−^ mice were fed with high-fat diets for eight weeks to induce atherosclerosis. Treatment with DHL for ten weeks resulted in a significant decrease in the serum levels of TC, TG, and LDL-C. Notably, DHL exhibited a distinct effect by significantly enhancing the levels of serum HDL-C, which sets it apart from simvastatin treatment. These results suggest that DHL treatment improves dyslipidemia in atherosclerotic mice. Regarding anti-atherosclerosis, DHL treatment led to a significant reduction in atherosclerotic plaque deposition in the vascular wall. In the arterial plaque stability assay, we observed a significant increase in collagen content and α-SMA expression (a marker for vascular smooth muscle cells) in the aortic root following DHL treatment, indicating greater stability of arterial plaques (Basatemur et al. 2019). The anti-atherogenic effects of DHL were more pronounced compared to simvastatin, possibly due to its ability to increase HDL-C levels.

Macrophage-mediated inflammation and lipid infiltration play crucial roles in the initiation, progression, and rupture of AS plaques. The imbalance between M1 and M2 macrophages is a key factor in driving inflammation (Deng et al. 2020). Clinical trials have demonstrated that inflammation inhibition can improve the outcomes for patients with atherosclerosis (Libby 2021b). The results of the present study indicated that DHL treatment significantly reduced the levels of IL-1β and TNF-α while increasing the level of IL-10 in both serum and aorta. Immunofluorescence staining also revealed that DHL treatment led to an increase in the number of M2 macrophages (CD163) and a decrease in the number of M1 macrophages (CD68) within the aorta. The activation of M2 macrophages is associated with the secretion of anti-inflammatory cytokines, such as IL-10, which can inhibit inflammation and enhance the stability of atherosclerotic plaques (Hou et al. 2023). Previous study has reported that costunolide (a sesquiterpene lactone isolated from costus root) can prevent inflammatory atherosclerosis by inhibiting the activation of NF-κB (Huang et al. 2023), which further demonstrates the anti-inflammatory effects of DHL from a complementary perspective. Thus, the anti-inflammatory effect of DHL is associated with its modulation of macrophage polarization, and this mechanism may involve the inhibition of toll-like receptors, as previously reported in the scientific literature (Wu et al. 2021).

Blood metabolomics analysis revealed that DHL treatment significantly reduced the relative abundance of sphingomyelins (SMs), phosphatidylcholines (PCs), and trimethylamine-N-oxide (TMAO). The metabolism of PCs by gut microbes produces trimethylamine (TMA), which is subsequently oxidized to generate TMAO (Wang et al. 2011). Therefore, elevated levels of circulating PCs are positively correlated with atherosclerosis, as TMAO serves as a significant atherogenic factor. Furthermore, PCs directly contribute to SMs synthesis (Iqbal et al. 2017), indicating that the decrease in SMs observed in the blood following DHL treatment may be attributed to this factor. The reduction in SMs could potentially lower the risk of atherosclerosis, as elevated levels of SMs have been associated with increased inflammation and lipid accumulation in the arterial walls (Slotte 2013). By reducing cellular SM levels, DHL may help alleviate inflammation and facilitate cholesterol efflux from the arteries, ultimately reducing the risk of atherosclerosis (Chakraborty et al. 2013).

Based on the in vivo study, DHL has the potential to exert athero-protective effects by enhancing cholesterol efflux from macrophage-derived foam cells. Cholesterol efflux assays in the present study demonstrated that DHL can dose-dependently promote cholesterol efflux from foam cells (both RAW264.7 cells and THP-1 cells), leading to a reduction in intracellular lipid droplets (visualized by oil red O staining) and TC levels. Additionally, DHL treatment significantly increased the protein expression of both ABCA1 and ABCG1 in the aorta and foam cells. The upregulation of these proteins further supports our hypothesis since ABCA1 and ABCG1 are critical receptors involved in initiating cholesterol efflux (Chistiakov et al. 2017). PPAR-γ/LXR, the key pathway known to regulate the expression of ABCA1/ABCG1, has been identified could enhance cholesterol efflux, thereby attenuating atherosclerosis (Zheng et al. 2021). Interestingly, DHL treatment also upregulated the protein expression of PPAR-γ and LXR in the aorta and foam cells, thus promoting the initiation of cholesterol efflux.

Numerous studies have consistently reported a significant upregulation of Toll-like receptors (TLRs) in macrophage-derived foam cells, highlighting their close association with cholesterol efflux, inflammation, and the progression of atherosclerosis (Edfeldt et al. 2002; Guo et al. 2015; Mogilenko et al. 2012; Stamatikos et al. 2019). Inhibiting TLRs not only suppresses inflammation but also activates transcription factors such as LXR or PPAR, which in turn facilitate cholesterol efflux (Li et al. 2020a, b; Vorkapic et al. 2015). The present study’s results demonstrate that DHL treatment reduces the protein expression of NF-κB and MyD88, leading to the inhibition of inflammation in atherosclerotic mice. Furthermore, DHL treatment decreases the protein expression of TLR2 and TLR4, with TLR2 being more abundantly expressed than TLR4 in macrophage-derived foam cells. DHL’s inhibitory effect on TLR2 is more pronounced in both arterial tissues and cells. Based on our findings, we postulated that TLR2 functions as the principal receptor mediating the anti-inflammatory and pro-cholesterol efflux effects of DHL.

To further verify this hypothesis, we conducted experiments using Pam3 CSK4 (a TLR2 agonist) and C29 (a TLR2 inhibitor) incubated with the cells. Interestingly, in the presence of Pam3 CSK4, DHL treatment still effectively increased the expression of PPAR-γ, LXR, ABCA1, and ABCG1, thereby promoting cholesterol efflux. Additionally, DHL treatment decreased the expression of NF-κB and improved the levels of cytokines (IL-1β, IL-10, and TNF-α). However, when TLR2 was blocked with C29, DHL treatment had no significant impact on related protein expression and inflammation, except for its ability to increase cholesterol efflux. In addition, the interaction between DHL and TLR2 is primarily mediated by hydrogen bonds with the oxygens of Lys 347. Binding to this amino acid residue has been shown to inhibit TLR2 activity, as previously reported in the literature (Grabowski et al. 2018). The binding energy of −7.753 kcal/mol suggests a highly stable binding between DHL and TLR2. Moreover, the unsaturated carbonyl group on the DHL molecule anchors I-κB kinase β, leading to the inhibition of the MyD88-dependent signal transduction pathway (Kim et al. 2020; Wang et al. 2017a, b). These results confirm that DHL exerts its anti-atherosclerotic effects through the TLR2 receptor, which can be attributed to its unique chemical structure.

## Conclusions

In conclusion, treatment with DHL greatly reduced blood lipid levels and decreased the level of atherosclerotic plaques in the aorta. Mechanistically, DHL effectively alleviates atherosclerosis by promoting cholesterol efflux and inhibiting inflammation through the TLR2/PPAR-γ/NF-κB signaling pathway, which suggests that DHL may serve as a new treatment strategy for atherosclerosis (As seen in Graphic abstract). The present study revealed that DHL exhibited anti-inflammatory properties and facilitated cholesterol efflux, leading to increased HDL-C levels. However, it was also observed that DHL effectively reduced serum LDL-C and TG levels. Further investigations are needed to explore the effect of DHL on hepatic lipid metabolism and the underlying mechanism of lowering TG and LDL-C.

## Data Availability

The original contributions presented in the study are included in the article/additional material, and further inquiries can be directed to the corresponding author.

## Abbreviations

AS: Atherosclerosis
LDL: Low-density lipoprotein
ox-LDL: Oxidized LDL
ABCA1: ATP-binding cassette A1
ABCG1: ATP-binding cassette G1
apoA-I: Apolipoprotein A-I
HDL: High-density lipoprotein
TLRs: Toll-like receptors
PPARs: Peroxisome proliferators-activated receptors
LXR: Liver X receptor
DHL: Dehydrocostus lactone
ApoE^−/−^: Apolipoprotein E-deficient
TG: Triglyceride
TC: Total cholesterol
H&E: Hematoxylin and eosin
α-SMA: Alpha-smooth muscle actin
IL: Interleukin
TNF-α: Tumor necrosis factor-α
GAPDH: Glyceraldehyde-3-phosphate dehydrogenase
MyD88: Myeloid differentiation factor 88
NF-κB: Nuclear factor kappa B
PC: Phosphatidylcholine
SM: Sphingomyelin
TMAO: Trimethylamine-N-oxide
